# Increased Heschl’s Gyrus Duplication in Schizophrenia Spectrum Disorders: A Cross-Sectional MRI Study

**DOI:** 10.3390/jpm11010040

**Published:** 2021-01-12

**Authors:** Tsutomu Takahashi, Daiki Sasabayashi, Yoichiro Takayanagi, Atsushi Furuichi, Mikio Kido, Tien Viet Pham, Haruko Kobayashi, Kyo Noguchi, Michio Suzuki

**Affiliations:** 1 Department of Neuropsychiatry, Graduate School of Medicine and Pharmaceutical Sciences, University of Toyama, Toyama 930-0194, Japan; ds179@med.u-toyama.ac.jp (D.S.); takayanagi-matsu@umin.net (Y.T.); ichi1031@med.u-toyama.ac.jp (A.F.); mikiokid@med.u-toyama.ac.jp (M.K.); tienke93@gmail.com (T.V.P.); harukodayoo@gmail.com (H.K.); suzukim@med.u-toyama.ac.jp (M.S.); 2 Research Center for Idling Brain Science, University of Toyama, Toyama 930-0194, Japan; 3 Arisawabashi Hospital, Toyama 939-2704, Japan; 4 Department of Radiology, Graduate School of Medicine and Pharmaceutical Sciences, University of Toyama, Toyama 930-0194, Japan; kyo@med.u-toyama.ac.jp

**Keywords:** magnetic resonance imaging, schizophrenia, schizotypal disorder, gyrification, superior temporal gyrus, neurodevelopment

## Abstract

Duplicated Heschl’s gyrus (HG) is prevalent in patients with schizophrenia and may reflect early neurodevelopmental anomalies. However, it currently remains unclear whether patients with schizotypal disorder, a prototypic disorder within the schizophrenia spectrum, exhibit a similar HG gyrification pattern. In this magnetic resonance imaging study, HG gyrification patterns were examined in 47 patients with schizotypal disorder, 111 with schizophrenia, and 88 age- and sex-matched healthy subjects. HG gyrification patterns were classified as single, common stem duplication (CSD), or complete posterior duplication (CPD). The prevalence of the duplicated HG patterns (CSD or CPD) bilaterally was higher in the schizophrenia and schizotypal groups than in healthy controls, whereas no significant difference was observed between the schizophrenia and schizotypal groups. Schizophrenia patients with the right CPD pattern had less severe positive symptoms, whereas the right single HG pattern was associated with higher doses of antipsychotic medication in schizotypal patients. The present study demonstrated shared HG gyrification patterns in schizophrenia spectrum disorders, which may reflect a common biological vulnerability factor. HG patterns may also be associated with susceptibility to psychopathology.

## 1. Introduction

Heschl’s gyrus (HG), which is also termed as transverse temporal gyrus, is a convolution on the superior temporal plane that hosts the primary auditory cortex (Brodmann area (BA) 41) [1,2]. Its gyral pattern markedly varies across individuals, with approximately 30% to 50% of healthy subjects having HG duplication, particularly on the right hemisphere [3,4,5]. In cases of HG duplication, cytoarchitectonic evidence suggests that the auditory koniocortex (BA41) is restricted to the anterior HG, whereas the posterior HG may correspond to BA42/22 as part of the associative auditory cortex [1,2,3,4,5]. This anatomical variant may reflect differences in cytoarchitectonic development during the gestational period [6,7], with duplicated HG potentially reducing HG functional activity during auditory processing [8]. However, it currently remains unclear whether patients with schizophrenia, who have an early neurodevelopmental pathology [9,10] and are characterized by altered volume/thickness and functional connectivity of HG [11,12,13], exhibit an altered HG gyrification pattern. In a recent magnetic resonance imaging (MRI) study, we demonstrated that HG duplication was prevalent in first-episode schizophrenia [14], whereas another MRI study in which HG duplication patterns in chronic schizophrenia were specifically examined failed to find significant differences [15]. Although gyrification patterns generally remain stable after birth in healthy subjects [6], this discrepancy may be partly explained by the potential impact of illness chronicity and/or antipsychotic medication on the morphology of the superior temporal plane [16].

Schizotypal disorder [17] or schizotypal personality disorder [18], a prototypical schizophrenia spectrum disorder without overt/sustained psychosis, may have biological, neurocognitive, and phenomenological similarities with schizophrenia as a common vulnerability factor [19,20]. Our recent whole brain analyses revealed that schizophrenia and schizotypal patients both exhibit increased cortical folding, which may be related to neural dysconnectivity due to aberrant neurodevelopmental processes [21], in diverse cortical regions [22,23]. Furthermore, schizotypal patients share decreased functional connectivity in the HG, which may reflect vulnerability to psychopathology [24,25], with patients with overt schizophrenia [26,27]. These previous findings suggest an altered HG gyrification pattern in schizotypal subjects, but no MRI studies to date have specifically examined it. Therefore, it currently remains unclear whether an altered HG gyrification pattern, if present, is specific to schizophrenia or is commonly observed in schizophrenia spectrum disorders.

The present MRI study compared HG gyrification patterns (single convolution, partially duplicated, or fully duplicated) between an expanded sample of schizophrenia patients of different illness stages (i.e., first-episode [14] and chronic), schizotypal disorder, and healthy controls. Based on biological commonalities among schizophrenia spectrum disorders as a vulnerability factor [16,19,20] and our previous findings of first-episode schizophrenia [14], we predicted that HG duplication will be more prevalent in the schizophrenia and schizotypal groups than in healthy controls. Based on the potential early neurodevelopmental pathology of schizophrenia [9,10] and the notion that the gyrification pattern generally remains stable after birth [6,7], we also predicted no influence of illness chronicity on the HG pattern in schizophrenia. Furthermore, we examined the relationship between HG patterns and other clinical variables (e.g., symptom severity and medication) in the patient groups.

## 2. Materials and Methods

### 2.1. Subjects

Subjects in the present cross-sectional study consisted of 47 patients with schizotypal disorder (or schizotypal personality disorder), 111 with schizophrenia, and 88 age- and sex-matched healthy subjects (Table 1). The patients were from our observational study (i.e., not from randomized clinical trials), whereas the controls were selected from our MRI database of healthy subjects on the basis of age and sex. They were all physically healthy at MRI and had no previous history of severe obstetric complications, serious head trauma, serious medical diseases (e.g., neurological illness, thyroid dysfunction, diabetes, and hypertension), steroid use, or substance abuse. All subjects were right-handed, Japanese (aged 14 to 46 years), and were screened for gross brain abnormalities by neuroradiologists at the time of scanning. Among 246 subjects, HG patterns in 64 schizophrenia and 64 control subjects were reported in our previous study [14], which demonstrated that HG duplication was more prevalent in first-episode schizophrenia. The study was conducted in accordance with the Declaration of Helsinki. The Committee on Medical Ethics of Toyama University approved the present study (No. I2013006) on 5 February 2014. After the purpose and procedures of the present study were fully explained, written informed consent was received individually from each study participant. When participants were <20 years old, written consent was also received from a parent/guardian.

Patients with schizotypal disorder and schizophrenia who met the ICD-10 research criteria [28] were recruited from the outpatient and inpatient clinics of the Department of Neuropsychiatry, Toyama University Hospital. Patients were diagnosed by experienced psychiatrists (M.K., T.T. and M.S.) in a structured interview (the Japanese version of Comprehensive Assessment of Symptoms and History [29]), in addition to clinical symptoms scored using the Japanese version of Scale for the Assessment of Negative and Positive Symptoms (SANS/SAPS [30,31]) and a detailed chart review. Cognitive and social functions were not systematically evaluated. The recruitment strategy and sample characteristics of our clinic-based schizotypal group who required clinical care for distress/issues stemming from their schizotypal features were described in detail previously [32,33]. All schizotypal patients fulfilled the DSM-IV criteria of the schizotypal personality disorder on Axis II, whereas 13 had previously experienced transient quasipsychotic episodes fulfilling a DSM Axis I diagnosis of brief psychotic disorder [18]. Although the risk of developing psychosis was previously reported to be higher in schizotypal patients than in the general population [34], none in the present study developed overt schizophrenia during the clinical follow-up period (mean = 3.0 ± 2.6 years). The schizophrenia group was divided into first-episode (illness duration ≤ 1 year (*N* = 48) or first psychiatric hospitalization (*N* = 16)) and chronic (illness duration ≥ 3 years (*N* = 41)) subgroups to examine the effects of illness chronicity. The medication status and other clinical data are summarized in Table 1.

Control subjects were recruited from the community (*N* = 29), hospital staff (*N* = 27), and university students (*N* = 32). They were screened using a questionnaire consisting of 15 items regarding their present/previous medical history and family histories of illness [35] and were excluded if they had any personal or family history of psychiatric illness among their first-degree relatives.

### 2.2. MRI Procedure

Brain MRI was performed using the 1.5-T Magnetom Vision (Siemens Medical System, Inc, Erlangen, Germany) at Toyama University Hospital. Three-dimensional gradient-echo sequence FLASH yielded a sagittal series of 160–180 contiguous T1-weighted slices with a thickness of 1.0 mm. Imaging parameters were as follows: time to echo = 5 ms, time repetition = 24 ms, flip angle = 40°, field of view = 256 mm, matrix size = 256 × 256, and voxel dimension = 1.0 × 1.0 × 1.0 mm.

### 2.3. Assessment of HG Gyrification Patterns

**Figure 1:**
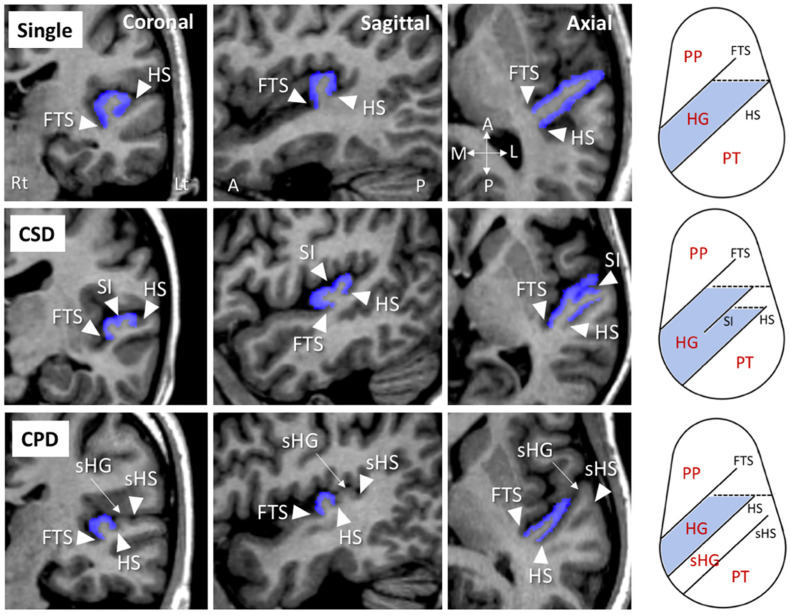
Coronal, sagittal, and axial views of sample MRI of Heschl’s gyrus (HG; colored in blue) and schematic drawings of the superior temporal surface on an axial view (right) of different gyrification patterns. A, anterior; CPD, complete posterior duplication; CSD, common stem duplication; FTS, first transverse sulcus; HS, Heschl’s sulcus; L, lateral; P, posterior; M, medial; PT, planum temporale; PP, planum polare; sHG, second Heschl’s gyrus; sHS, second Heschl’s sulcus; SI, sulcus intermedius.

As described in detail previously [14], HG patterns on each hemisphere were classified as follows: single HG, common stem duplication (CSD), and complete posterior duplication (CPD) [2,3,4,5]. Anatomical landmarks for this classification were facilely identified on coronal, axial, and sagittal views simultaneously displayed using Dr. View software (Infocom, Tokyo, Japan) (Figure 1). Briefly, a single HG pattern had no duplication (i.e., one HG per hemisphere), whereas the CPD pattern was defined by completely separate gyri (two (*N* = 134) or three (*N* = 6) gyri per hemisphere in this study). The CSD pattern was characterized by a gyrus that was partially split by the sulcus intermedius (SI), which forms a “heart-shaped” HG. Eight subjects (6.5%) in the present study who had a separate HG posterior to the HG with partial duplication were considered to have the CSD pattern.

All pattern classifications were performed by one rater (T.T.) without knowledge of the subjects’ identity (e.g., sex and diagnosis). The HG pattern in a subset of 15 randomly selected brains (30 hemispheres) was assessed independently by two raters (T.T. and D.S.), and each HG pattern was then reclassified after at least 4 weeks by T.T., who was blinded to the first HG classification; inter- (T.T. and D.S.) and intrarater reliabilities (Cronbach’s α) were 0.83 and 1.00, respectively.

### 2.4. Statistical Analysis

A one-way analysis of variance (ANOVA) or the χ^2^ test was performed to assess the significance of group differences in clinical/demographic data.

The HG pattern distribution on each hemisphere was compared across the groups by the χ^2^ test. As the HG duplication was more prevalent in the patient groups regardless of its subtype, the odds ratio was calculated to estimate the association between HG duplication (CSD or CPD) and relative risk of developing schizophrenia/schizotypal disorder. Analysis of covariate (ANCOVA) was used to examine the potential contribution of HG pattern to total SANS/SAPS scores, with the HG type as an independent variable and the medication dose/duration as covariates. Significant effects yielded in ANCOVA were then analyzed using post hoc Newman–Keuls tests. The relationship between the HG gyrification pattern and other clinical variables (i.e., onset age and duration of illness (only for the schizophrenia group), and medication status (dose and duration)) was assessed by nonparametric Kruskal–Wallis tests due to the non-normal distribution of these variables (tested by Kolmogorov–Smirnov test). The relationship between the HG type and SAPS score in schizophrenia was also tested by the Kruskal–Wallis test because of the skewed distribution of the score (Kolmogorov–Smirnov test, *p* = 0.018). In order to estimate potential interaction effects of illness stages (first-episode vs. chronic) and other factors (e.g., medication), each subgroup was also tested independently. To test for potential sampling bias, an independent contribution of demographic variables (age, sex, height, education, and parental education) on HG pattern was investigated using stepwise regression analysis. The significance of differences was defined as *p* < 0.05.

## 3. Results

### 3.1. Demographic and Clinical Data

Groups were matched for age, sex, and parental education, but control subjects had a higher education level than patients with either disorder (Table 1). As expected, schizophrenia patients had significantly more severe symptom ratings (SANS/SAPS) and higher medication doses than schizotypal patients.

### 3.2. HG Pattern Distributions

**Figure 2:**
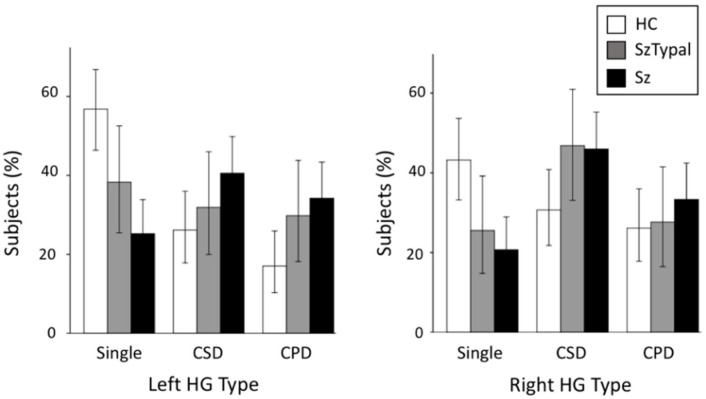
Distribution of Heschl’s gyrus (HG) gyrification patterns in schizophrenia (Sz), schizotypal (SzTypal), and healthy control (HC) groups. Error bars show 95% confidence intervals. CPD, complete posterior duplication; CSD, common stem duplication.

The schizophrenia (left, χ^2^ = 20.56, *p* < 0.001; right, χ^2^ = 11.84, *p* = 0.003) and schizotypal (left, χ^2^ = 4.20, *p* = 0.040; right, χ^2^ = 4.09, *p* = 0.043) groups had a significantly higher prevalence of duplicated HG patterns bilaterally (i.e., CSD or CPD) than healthy controls, whereas no significant differences were observed in the HG pattern between the schizophrenia and schizotypal groups (χ^2^ < 2.73, *p* > 0.098) (Table 2, Figure 2). When we examined subjects with HG duplication only, no group difference was noted in HG patterns (CSD vs. CPD; χ^2^ < 0.66, *p* > 0.416). The odds ratio of HG duplication was 3.90 (left, 95% confidence interval (CI) = 2.14 to 7.12) and 2.91 (right, 95% CI = 1.56 to 5.42) for schizophrenia, and 2.12 (left, 95% CI = 1.03 to 4.37) and 2.22 (right, 95% CI = 1.02 to 4.83) for schizotypal disorder.

HG patterns were also compared between the first-episode and chronic subgroups of schizophrenia, but no significant differences were observed (left, χ^2^ = 0.00, *p* = 0.998; right, χ^2^ = 2.23, *p* = 0.328).

Sex did not significantly affect HG patterns (χ^2^ < 2.61, *p* > 0.112), whereas HG duplication (i.e., CSD or CPD) was more frequent in the right hemisphere (χ^2^ = 4.77, *p* = 0.029) when all diagnostic groups were combined.

Stepwise analysis of demographic variables using the entire sample revealed that the HG pattern was predicted by height (beta = 0.160, t = 2.45, *p* = 0.015) and education (beta = −0.152, t = −2.32, *p* = 0.021) only for the right hemisphere (adjusted *R*^2^ = 0.031). For validation purposes, we then assessed the relationship between these variables and right HG pattern using the Kruskal–Wallis test; however, only the education level had a trend-level relationship with the HG pattern (*H* = 5.878, *p* = 0.053).

### 3.3. Relationship between the HG Pattern and Clinical Variables

The right HG pattern significantly affected the total SAPS score in the schizophrenia group (ANCOVA, *F* (2, 99) = 4.47, *p* = 0.014), with a lower score being observed in patients with the CPD pattern (mean = 19.8, SD = 15.9) than in those with the CSD (mean = 30.0, SD = 22.6; *p* = 0.038) or single (mean = 32.5, SD = 21.9; *p* = 0.027) pattern. This relationship was replicated using a nonparametric Kruskal–Wallis test (*H* = 6.61, *p* = 0.037) and post hoc pairwise comparisons (CPD < CSD (*p* = 0.033) or single (*p* = 0.022)). Furthermore, subgroup analysis demonstrated that this relationship was specific to first-episode schizophrenia (ANCOVA, *F* (2, 60) = 3.78, *p* = 0.028; Kruskal–Wallis test, *H* = 7.78, *p* = 0.020). The chronic schizophrenia patients with left single HG (mean = 37.9, SD = 13.2) had a higher SAPS score than those with left CSD (mean = 26.4, SD = 26.0; *p* = 0.011) or left CPD (mean = 24.7, SD = 13.1; *p* = 0.053) (Kruskal–Wallis test, *H* = 6.70, *p* = 0.035), but this relationship was not observed using ANCOVA with medication dose and duration as covariates (*F* (2, 34) = 0.66, *p* = 0.526).

Onset age, illness duration, and medication were not related to the HG pattern in the schizophrenia group as a whole (*H* < 4.81, *p* > 0.090). However, based on subgroup analyses, patients with left CPD received a lower medication dose (mean = 4.9 mg/day, SD = 3.1) than those with single HG (mean = 15.1 mg/day, SD = 10.0; *p* = 0.005) or CSD (mean = 11.4 mg/day, SD = 10.1; *p* = 0.064) only in the chronic subgroup (Kruskal–Wallis test, *H* = 8.31, *p* = 0.016).

No relationship was observed between the HG pattern and symptom severity in the schizotypal group (*F* (2, 40) < 1.46, *p* > 0.244). However, the right HG pattern significantly affected the medication dose (*H* = 5.71, *p* = 0.017). Schizotypal patients with the single HG pattern (mean = 8.4 mg/day, SD = 8.3) were characterized by a higher medication dose than in those with the CSD (mean = 3.6 mg/day, SD = 4.1) or CPD (mean = 3.4 mg/day, SD = 4.0) pattern.

## 4. Discussion

The present study of HG gyrification patterns in the schizophrenia spectrum demonstrated that the prevalence of HG duplication was higher in schizophrenia patients regardless of the illness stage and that schizotypal disorder patients share a similar HG gyrification pattern. Furthermore, the HG pattern was associated with the severity of psychotic symptoms in schizophrenia patients and with the necessity for a high dose of antipsychotics in both patient groups. The present study supports the gross morphology of the HG reflecting a neurobiological basis for vulnerability factors commonly observed in the schizophrenia spectrum.

As predicted, HG duplication (CSD or CPD) was more prevalent in schizophrenia patients in both the first-episode and chronic stages, and was not related to illness duration. However, previous longitudinal MRI studies demonstrated active gray matter atrophy in the superior temporal region, particularly during the early illness stages of schizophrenia [36,37]. These previous and present studies support the altered sulcogyral pattern in schizophrenia being a stable trait marker associated with neurodevelopmental anomalies [38,39], and the altered HG pattern and atrophy of superior temporal structures having independent mechanisms [14]. Although Hubl et al. [15] reported that the prevalence of duplicated HG was only slightly higher in hallucinating schizophrenia patients than in healthy controls, this negative result may be partly attributed to the small sample size (13 patients and 13 controls) and their anatomical definition that treated the CSD pattern as a variant of single HG. As we demonstrated that HG duplication was more prevalent in schizophrenia regardless of its subtype (CSD or CPD), the schizophrenia cohort by Hubl et al. [15] must have had a higher prevalence of HG duplication according to the traditional HG classification (single vs. duplicated (CSD or CPD)).

One of the main results of the present study was that schizotypal patients considered to share vulnerability-associated biological characteristics with schizophrenia [20] exhibited an altered HG pattern similar to that in patients with schizophrenia. The HG gyrification pattern is thought to reflect neurodevelopmental processes during gestation [7]. Thus, the present results are consistent with previous studies demonstrating shared alterations in neurodevelopmental markers, such as malformation of the adhesio interthalamica [40,41] and widespread cortical hypergyria [23], in schizophrenia spectrum disorders. Conversely, an altered orbitofrontal sulcogyral pattern was specific to schizophrenia and not observed in schizotypal patients [42]. This may support the emergence of overt/sustained psychosis being mitigated by a less deviant frontal neurodevelopmental change in the milder form of schizophrenia spectrum disorders, which may explain the less severe prefrontal atrophy [33,43] and frontal–striatal–temporal dysconnectivity [44] in schizotypal patients than in schizophrenia patients. Collectively, these findings and the present study support the hypothesis that temporal changes underlie vulnerability to schizophrenia, whereas latent dysfunction becomes clinically apparent as the emergence of psychotic symptoms due to additional frontal pathology [45].

The present study replicated the findings of our first-episode schizophrenia study [14] in an expanded schizophrenia group with differing illness stages in that a relationship was observed between the CPD pattern and less susceptibility to psychotic symptoms. Furthermore, our study suggested that the single HG pattern in schizophrenia is associated with poor treatment response (i.e., prolonged positive psychotic symptoms and need for higher doses of antipsychotics) at chronic stages. We also suggested that schizotypal patients with the single HG pattern need higher doses of antipsychotics than those with other HG patterns to control their severe symptoms (e.g., distress and/or transient quasipsychotic symptoms). As the brain gyrification pattern may reflect underlying neural connectivity [21], our study is partly consistent with functional neuroimaging studies in which schizophrenia [26] and schizotypal [27] patients both exhibited abnormal connectivity involving the superior temporal plane, which may underlie the vulnerability to psychopathology [24,25,46]. However, the functional role of the HG gyrification pattern remains controversial; duplicated HG is associated with reduced HG activity during word-listening tasks [8] and learning disabilities [4,47], whereas individuals with HG duplications exhibited advanced auditory skills with an expanded activation area [48,49]. Therefore, further studies are needed to elucidate the functional significance of gross HG gyrification patterns under both normal and pathological conditions.

Several limitations need to be addressed. First, it was not possible to reliably assess group differences in the prevalence of HG multiplications (i.e., ≥3 gyri or CSD with independent second HG) because only 14/246 (5.7%) subjects (eight schizophrenia, three schizotypal, and three control subjects) had these patterns. As HG multiplications function in advanced auditory processing [48,49], further studies are needed using a larger cohort to establish whether this rare HG pattern is associated with the pathophysiology of schizophrenia spectrum disorders. Regarding the sample issue, the smaller sample size of healthy controls (*N* = 88) compared with schizophrenia (*N* = 111) and their high education level may have biased our findings. The lower prevalence of the single HG pattern in our control subjects (left, 56.8%; right, 43.2%) than in previous post mortem or MRI studies (up to 75%) [3] may be partly due to these sampling problems. Multiplicity of statistical analyses should be also noted. Second, as described above, the functional significance of HG gyrification patterns was not directly demonstrated in the present morphological MRI study. Moreover, cognitive functioning was not systematically evaluated in schizophrenia spectrum patients. The HG corresponds to the primary auditory cortex, but also plays a role in the processing of other cognitive domains, including memory and emotion [50,51]. Therefore, future multimodal neuroimaging studies are needed to investigate the function/connectivity of the brain in different HG patterns and its role, particularly in cognitive functioning, in the schizophrenia spectrum. Lastly, atypical sulcogyral patterns in other brain regions (e.g., the orbitofrontal cortex) have also been demonstrated in neuropsychiatric disorders, such as bipolar [52] and autism spectrum [53] disorders, suggesting that the sulcogyral phenotype represents a transdiagnostic trait marker. Therefore, the disease specificity of the present results on HG gyrification patterns warrants further study.

## 5. Conclusions

This MRI study demonstrated that the prevalence of HG duplications bilaterally was significantly higher in schizotypal patients than in healthy controls. This was similarly observed in patients with overt schizophrenia. In contrast to active gray matter changes in the superior temporal plane during the early stages of schizophrenia [12], the HG gyrification pattern was independent of the illness stage of schizophrenia, but it was partly associated with susceptibility to psychotic symptoms in schizophrenia spectrum disorders. Therefore, the HG gyrification pattern may be a trait marker commonly observed in schizophrenia spectrum disorders, which represents vulnerability to psychopathology due to early neurodevelopmental abnormalities. The present cross-sectional study should be interpreted with caution due to the limitations described above, in addition to the hypothesis that brain gyrification can change over time during the course of schizophrenia [54]. However, our study supports the notion that gross brain morphologic features (e.g., cortical folding) can aid in the classification of neuropsychiatric diseases (e.g., diagnosis and prediction of treatment response) in combination with multimodal neuroimaging data [16]. Furthermore, as clinical high-risk individuals with later psychosis onset may be characterized by more prominent gross brain characteristics [16], the HG pattern in a high-risk cohort in future studies should be assessed to test its possible role as a predictive marker of psychosis, which may lead to the development of specific and targeted preventive strategies [55].

## Figures and Tables

**Table 1:** Demographic/clinical parameters and brain measurements of subjects.

	C	SzTypal	Sz	Group Comparisons
Male/female	49/39	29/18	59/52	Chi squared = 0.98, *p* = 0.613
Age (years)	24.1 ± 6.0	25.0 ± 5.4	25.8 ± 5.4	*F* (2, 243) = 2.38, *p* = 0.095
Height (cm)	166.3 ± 7.8	165.9 ± 8.7	164.5 ± 8.0	*F* (2, 243) = 1.34, *p* = 0.264
Education (years)	15.7 ± 3.0	13.1 ± 2.0	13.5 ± 2.0	*F* (2, 243) = 26.23, *p* < 0.001; Sz, SzTypal < C
Parental education (years) ^1^	13.0 ± 2.3	12.3 ± 1.7	12.5 ± 2.0	*F* (2, 234) = 1.81, *p* = 0.166
Age of onset (years)	-	-	22.2 ± 4.7	-
Duration of illness (years)	-	-	3.6 ± 4.1	-
Dose of medication (HPD equivalent, mg/day)	-	4.8 ± 5.7	10.1 ± 8.8	*F* (1, 156) = 14.64, *p* < 0.001; SzTypal < Sz
Duration of medication (years)	-	1.5 ± 3.0	2.7 ± 3.6	*F* (1, 156) = 3.70, *p* = 0.056
Medication type (typical/atypical/mixed) ^2^	-	14/26/0	40/65/4	Fisher’s exact test, *p* = 0.636
Total SAPS scores ^3^	-	16.0 ± 9.2	27.2 ± 20.9	*F* (1, 147) = 11.86, *p* < 0.001; SzTypal < Sz
Total SANS scores ^3^	-	41.9 ± 21.7	49.8 ± 22.8	*F* (1, 147) = 3.93, *p* = 0.049; SzTypal < Sz

Values represent means ± standard deviations (SDs) unless otherwise stated. C, controls; HPD, haloperidol; SANS, scale for the assessment of negative symptoms; SAPS, scale for the assessment of positive symptoms; Sz, schizophrenia; SzTypal, schizotypal disorder. ^1^ Data missing for one control, four SzTypal, and four Sz subjects. ^2^ Seven SzTypal patients were antipsychotic-naïve. Two Sz patients were antipsychotic-free at scanning. ^3^ Data missing for two SzTypal and seven Sz patients.

**Table 2:** Gyrification pattern of Heschl’s gyrus (HG) in subjects.

**Healthy Controls**					
		Right HG pattern (*N* (%))			
		Single	CSD	CPD	Total
Left HG pattern (*N* (%))	Single	25 (28.4)	13 (14.8)	12 (13.6)	50 (56.8)
	CSD	8 (9.1)	9 (10.2)	6 (6.8)	23 (26.1)
	CPD	5 (5.7)	5 (5.7)	5 (5.7)	15 (17.0)
	Total	38 (43.2)	27 (30.7)	23 (26.1)	88 (100.0)
**Schizotypal Disorder**					
		Right HG pattern (*N* (%))			
		Single	CSD	CPD	Total
Left HG pattern (*N* (%))	Single	7 (14.9)	5 (10.6)	6 (12.8)	18 (38.3)
	CSD	2 (4.3)	10 (21.3)	3 (6.4)	15 (31.9)
	CPD	3 (6.4)	7 (14.9)	4 (8.5)	14 (29.8)
	Total	12 (25.5)	22 (46.8)	13 (27.7)	47 (100.0)
**Schizophrenia**					
		Right HG pattern (*N* (%))			
		Single	CSD	CPD	Total
Left HG pattern (*N* (%))	Single	11 (9.9)	11 (9.9)	6 (5.4)	28 (25.2)
	CSD	8 (7.2)	25 (22.5)	12 (10.8)	45 (40.5)
	CPD	4 (3.6)	15 (13.5)	19 (17.1)	38 (34.2)
	Total	23 (20.7)	51 (45.9)	37 (33.3)	111 (100.0)

CSD, common stem duplication; CPD, complete posterior duplication.

## Data Availability

The data presented in this study are available on request from the corresponding author. The data are not publicly available since we do not have permission to share the data.
